# Uniparental isodisomy of chromosome 1 results in glycogen storage disease type III with profound growth retardation

**DOI:** 10.1002/mgg3.634

**Published:** 2019-03-27

**Authors:** Emanuela Ponzi, Viola Alesi, Francesca R. Lepri, Silvia Genovese, Sara Loddo, Mafalda Mucciolo, Antonio Novelli, Carlo Dionisi‐Vici, Arianna Maiorana

**Affiliations:** ^1^ Division of Metabolism, Department of Pediatrics Specialties Bambino Gesù Children's Hospital Rome Italy; ^2^ Medical Genetics Unit, Medical Genetics Laboratory Bambino Gesù Children's Hospital Rome Italy

**Keywords:** genomic imprinting, glycogen storage disease type III, severe growth retardation, uniparental isodisomy

## Abstract

**Background:**

Glycogen storage disease type III (GSDIII) is caused by mutations of *AGL* gene with debranching enzyme deficiency. Patients with GSDIII manifest fasting hypoglycemia, hepatomegaly, hepatopathy, myopathy, and cardiomyopathy. We report on an 18‐year‐old boy with a profound growth retardation (<3 *SD*) besides typical clinical features of GSDIII, whereby endocrinological studies were negative.

**Methods and Results:**

Molecular analysis of *AGL* gene revealed the homozygous reported variant c.3903_3904insA. Since discordant results from segregation studies showed the carrier status in one parent only, SNP array and short tandem repeats analyses were performed, revealing a paternal disomy of chromosome 1 (UPD1).

**Conclusion:**

This study describes the first case of GSDIII resulting from UPD1. UPD can play an important role even in case of imprinted genes. *DIRAS3 *is a maternally imprinted tumor suppressor gene, located on chromosome 1p31, and implicated in growth and oncogenesis. It can be speculated that *DIRAS3* overexpression might have a role in the severe short stature of our patient. The study emphasizes the importance of parental segregation analysis especially in patients with recessive conditions to look for specific genetic causes of disease and to estimate properly the risk of family recurrence.

## 1 INTRODUCTION

Glycogen storage disease type III (GSDIII; OMIM #232400), also known as Cori‐Forbes disease, is a rare autosomal recessive inborn error of glycogen degradation with an incidence of 1:100,000 (Sentner et al., 2016) and a variable clinical severity, affecting primarily liver, heart, and skeletal muscle (Kishnani et al., 2010). GSDIII is caused by mutations in the AGL (*610860) gene with consequent deficiency of the glycogen debranching enzyme (GDE; EC no. 3.2.1.33 and 2.4.1.25, UniProt P35573), which has two independent catalytic activities, 4‐alpha‐glucanotransferase and amylo‐1,6‐glucosidase (Ding, Barsy, Brown, Coleman, & Chen, 1990). Phenotypically, patients can be further classified into having GSDIIIa (±85%), with involvement of the liver, heart, and skeletal muscle, or GSDIIIb (±15%), in which only the liver is affected (Laforêt, Weinstein, & Smit, 2012). Human *AGL* gene is located on chromosome 1p21 and consists of 35 exons spanning ~85 kb of genomic DNA (Yang, Ding, Enghild, Bao, & Chen, 1992). Six mRNA isoforms are present, differing in the 5' untranslated region and tissue distribution. The major mRNA isoform present in both the muscle and liver encodes a protein consisting of 1,532 amino acid residues (Kishnani et al., 2010). Clinical manifestations of GSDIII include hepatomegaly, hypertransaminasemia, fasting intolerance with ketotic hypoglycemia, growth retardation, and in many patients, progressive myopathy and cardiomyopathy. Frequently, hepatomegaly tends to resolve spontaneously. In patients with GSDIIIa, cardiomyopathy may become predominant in adults. Patients are treated with high protein diet (Derks & Smit, 2015) or more recently with ketogenic diet, particularly in case of cardiomyopathy (Valayannopoulos et al., 2011). To date, about 240 different disease‐causing *AGL* mutations are recorded in the Human Gene Mutation Database (http://www.hgmd.org) and other references. The majorities of GSDIII cases are caused by missense/nonsense, deletion, insertion, and splicing mutations.

Here, we report on the first proband in whom GSDIII results from paternal uniparental disomy (UPD) of chromosome 1, uncovered by discordant segregation study results.

## 2 MATERIALS AND METHODS

### 2.1 Case report

**Figure 1:**
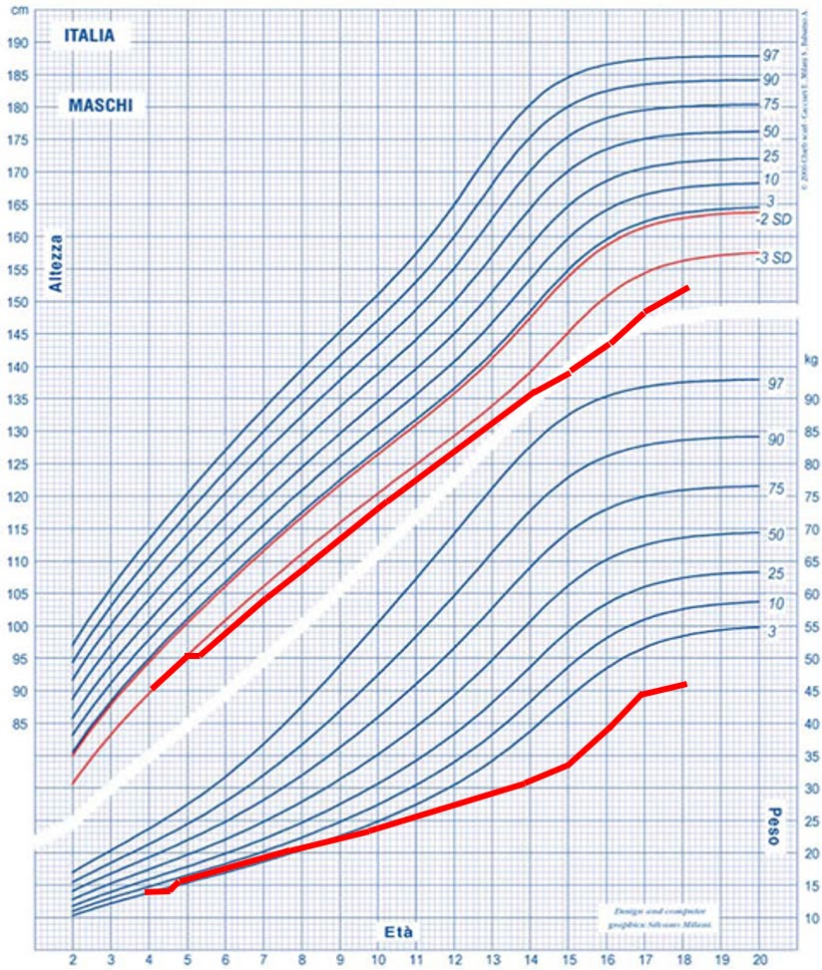
Growth chart. The chart displays the severe growth retardation of the patient over time

The patient born from non‐consanguineous Italian parents was referred to our Metabolic Unit at the age of 2 years and 10 months for hepatomegaly. The child presented with enlarged liver, hypertransaminasemia, and severe failure to thrive. Patient was evaluated with clinical examination and biochemical workup (including liver function tests, liver and muscle enzymes, metabolic and endocrine tests). Enzymatic activity of amylo‐1,6‐glucosidase was low (0.19 U/Hb g, normal value >1.31), suggesting a GSDIII. Over the years, he showed a severe harmonic growth retardation, with a height <3 *SD* at the age of 18 years (Figure 1), whereas the target height was at 10th–25th percentile. Despite retarded bone age, thyroid hormones assay and pituitary axis evaluation with multiple growth hormone stimulation tests, IGF1 generation test (Table 1), and cerebral MRI were unremarkable. Puberty started spontaneously at the age of 16 years. The patient was treated with a normocaloric high protein diet, supplemented with calcium and vitamin D (Derks & Smit, 2015) to promote gluconeogenesis, muscle mass growth, and bone mineralization. No other therapy potentially affecting growth was administered.

**Table 1:** Endocrinological evaluations of the patient

Age (years)	5^1/12^	5^4/12^	8^2/12^	11^11/12^
Bone age^a^ (years)			5	10
FT4 (ng/dl)			1.13	1.23
TSH (µU/ml)			4.94	3.51
IGF1 (ng/ml)			128	132
Arginine testing^b^ (GH peak ng/ml)	6			
Clonidine testing for GH^b^ (GH peak ng/ml)		14		
IGF1 generation test (ng/ml)			128‐>188	
GHRH + arginine test^c^^,^ d (GH peak ng/ml)				>40

a According to Greulich and Pyle.

b Cutoff 10 ng/ml.

c After priming with testosterone enanthate.

d Cutoff 20 ng/ml.

### 2.2 Analysis of genomic DNA

A written informed consent was obtained from the patient and parents for molecular analysis. Genomic DNA was isolated from whole peripheral blood using QIA symphony magnetic‐bead technology (www.qiagen.com). The entire coding region and flanking intronic sequence of *AGL* gene (NG_012865.1) were analyzed by Sanger sequencing using standard protocols.

### 2.3 Single nucleotide polymorphism array

Illumina BeadChip 850K platform (Illumina, San Diego, CA) was used for single nucleotide polymorphism (SNP) array analysis, according to the manufacturer's instructions, and results were analyzed by Bluefuse Multi Software (BlueGnome, Cambridge, UK).

### 2.4 Short tandem repeats analysis for UPD

Uniparental disomy of chromosome 1 was evaluated in patient analyzing four informative short tandem repeats (STR) polymorphic markers (D1S450, D1S234, D1S2878, and D1S213). STR markers were amplified with fluorescently labeled oligonucleotides from the ABI Prism Linkage Mapping Set Version 2.5 (Applied Biosystems, Warrington, UK), as recommended by the manufacturer. Electrophoretic analysis was performed on an ABI Prism 3130 Genetic Analyzer (Life Technologies) with Performance Optimized Polymer 7 using the Genescan software (Life Technologies).

## 3 RESULTS

### 3.1 *AGL* gene mutational analysis

DNA targeted sequencing of *AGL* gene revealed a homozygous single nucleotide insertion c.3903_3904insA (p.N1304*fs*Ter7), located in the exon 30. The familial segregation study showed the heterozygous mutation only in the father, with the mother resulting wild type.

### 3.2 SNP array and STR analysis

**Figure 2:**
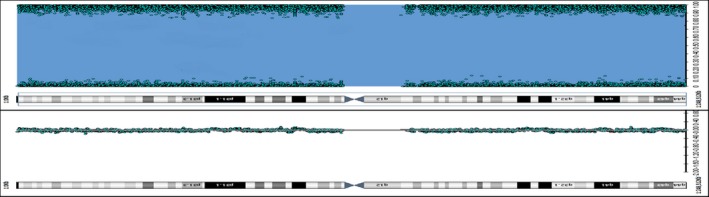
SNP array. SNP probes profile showed a long contiguous stretch of homozygosity encompassing the whole chromosome 1. SNP: single nucleotide polymorphism array

To test the presence of deletions or the occurrence of UPD, SNP array was performed resulting in a long contiguous stretch of homozygosity encompassing the whole chromosome 1, in which the *AGL* gene is located (1p21) (Figure 2). No deletions or duplications were observed. This result was consistent with the homozygous status of the *AGL* mutation. The results’ integration from Sanger sequencing, SNP array, and STR segregation study were suggestive of paternal isodisomy of chromosome 1.

## 4 DISCUSSION

**Figure 3:**
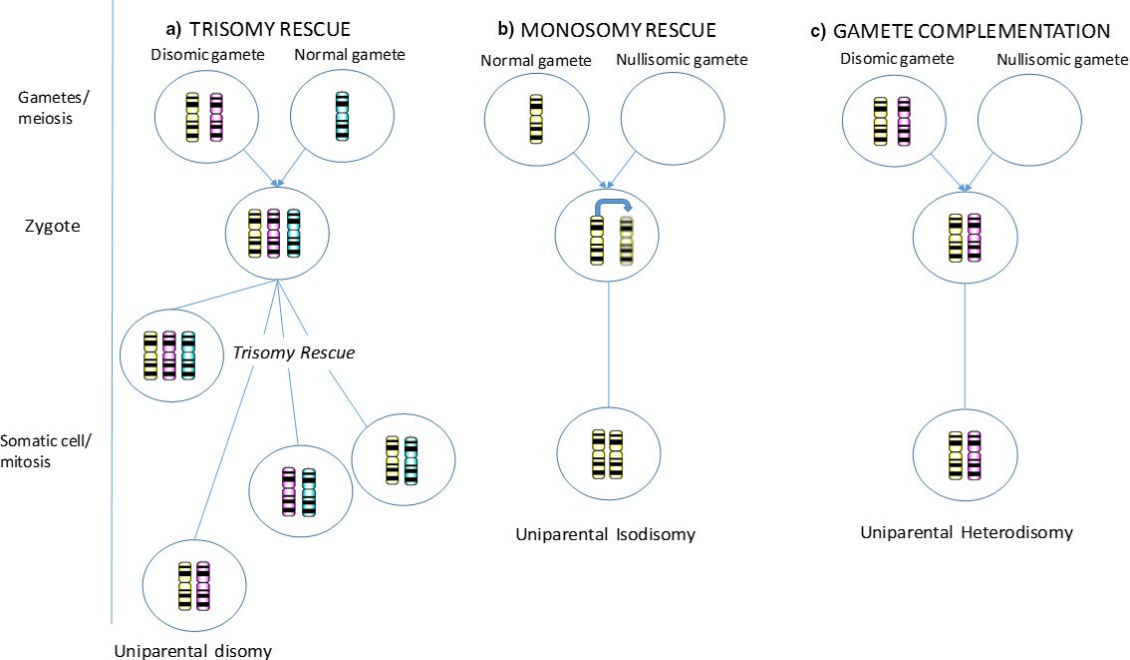
Mechanisms of UPD formation. (a) Mechanism of trisomy and trisomy rescue; (b) Mechanism of monosomy rescue; (c) Mechanism of gamete complementation. UPD: uniparental disomy

In this study, the molecular genetic basis of an Italian GSDIII patient was investigated using Sanger sequencing, SNP array, and STR analysis. The clinical features of the patient were consistent with GSDIII; therefore, Sanger sequencing was performed and an apparently homozygous single nucleotide insertion (c.3903_3904insA) was observed. This variant has been previously identified in compound heterozygous state with another single nucleotide insertion in a GSDIIIa Ashkenazi Jewish patient (Parvari, Shen, Hershkovitz, Chen, & Moses, 1998) and causes frameshift and stop codon formation at the amino acid position 1304. The results of segregation analysis were discordant to the homozygous status of the proband, with only one carrier between the parents. Mechanisms to explain our genetic results could be searched in a large deletion encompassing a portion or the entire *AGL* gene in the other allele or in the occurrence of UPD in which the proband inherits both copies of chromosome 1 from one parent. To test these two hypotheses, additional investigations with SNP array and STR analysis were performed and a complete paternal isodisomy of the whole chromosome 1 was successfully identified. This is the first report of GSDIII caused by uniparental inheritance. The frequency of UPD is estimated to be approximately 1/3,500–1/5,000 (Liehr, 2010) and its potential to unmask recessive alleles has been described for several diseases (Robinson, 2000). UPD can result from errors in chromosome segregation during gametogenesis and zygote formation. Meiotic nondisjunction results either in one hyperaploid gamete with two homologous chromosomes (anaphase I)/two sisters chromatids (anaphase II), and one hypoaploid gamete. Fecundation of a hyperaploid gamete results in a trisomic zygote; therefore, one of the three copies of the same chromosome can be lost, leading to UPD (trisomy rescue) (Figure 3a). Fecundation of a hypoaploid gamete could be solved by a complete replication of the monosomic chromosome, resulting in UPD (monosomy rescue) (Figure 3b). Fecundation of a hyperaploid with a hypoaploid gamete results in a zygote with UPD (gamete complementation) (Figure 3c) (Zneimer, 2014). In a trisomy rescue, the meiotic crossing‐over which precedes the two homologous/chromatids nondisjunction event (anaphase) always leads to the presence of both regions of heterodisomy and isodisomy on the involved chromosome. On the contrary, in a monosomy rescue, a single chromosome is post‐zygotically replicated, resulting in an identical pair of chromosomes (isodisomy) (Conlin et al., 2010). Since our proband showed a complete isodisomy and no evidence of paternal recombination, we hypothesized that UPD was more likely due to the complete duplication of the paternal chromosome 1 in a monosomy rescue mechanism. Definitely, the isodisomy unmasked the recessive mutation of *AGL*, leading the pathogenic variant to a homozygous state. The patient phenotype was compatible with GSDIII diagnosis, although clinical history was characterized by a profound growth delay, without hormone deficiencies. UPD can play an important role even in case of imprinted genes. Particularly, *DIRAS3* (*605193) is a maternally imprinted tumor suppressor gene, located in 1p31 and implicated in growth and ovarian, breast (Yu et al., 1999) and follicular thyroid cancer (Weber et al., 2005). To date, several cases of UPD1 have been described (Turner et al., 2007), some of paternal and others of maternal origin. All of them were identified through the detection of homozygosity for an autosomal recessive disorder in discordance with the segregation analysis. Other two cases were incidentally identified during genome‐wide linkage analysis (Field, Tobias, Robinson, Paisey, & Bain, 1998; Miyoshi et al., 2001) and further two cases have been described with a phenotype that could not be attributed to a known recessive condition (Chen et al., 1999; Röthlisberger et al., 2001). Particularly, the karyotype of a female with paternal UPD1 (Chen et al., 1999) presenting with myopathy, infertility and short stature, showed the presence of two isochromosomes 1, which likely have arisen from an abnormal division following chromosome 1 monosomy. In this case, although other potential mechanisms responsible for the phenotype may be hypothesized (unrecognized recessive condition, unrecognized microdeletion associated to isochromosome formation), some of the clinical features, as short stature and sterility, may be due to the imprinting of *DIRAS3*, since its overexpression may have a role in reducing growth and ovarian function (Xu et al., 2000). Indeed, transgenic mice overexpressing *DIRAS3* have been reported to have significantly lower body weight than controls (Xu et al., 2000). Furthermore, hypomethylation with consequent overexpression of *DIRAS3* has been described in a patient with severe growth retardation and clinical diagnosis of Silver–Russel syndrome (Fuke et al., 2013). Therefore, it could be speculated that *DIRAS3 *overexpression could have a pathogenetic role in the severe short stature of our patient with paternal UPD1.

In conclusion, we described the first case of GSDIII resulting from UPD1. The application of SNP array and STR analysis revealed that the apparent homozygous condition of the patient was due to paternal UPD1. The study emphasizes the importance of parental segregation studies especially in patients with recessive conditions to look for specific genetic causes of disease and to estimate properly the risk of family recurrence. Indeed, in case of UPD associated to recessive conditions, the recurrence risk can be neglectable if only one parent carries the mutation.

## CONFLICT OF INTEREST

All the authors have nothing to disclose.
